# Improvement of Antitumor Therapies Based on Vaccines and Immune-Checkpoint Inhibitors by Counteracting Tumor-Immunostimulation

**DOI:** 10.3389/fonc.2018.00006

**Published:** 2018-01-26

**Authors:** Paula Chiarella, Mónica Vermeulen, Daniela R. Montagna, Pablo Vallecorsa, Ariel Ramiro Strazza, Roberto P. Meiss, Oscar D. Bustuoabad, Raúl A. Ruggiero, Richmond T. Prehn

**Affiliations:** ^1^Department of Experimental Oncology, Instituto de Medicina Experimental, Academia Nacional de Medicina (CONICET), Academia Nacional de Medicina de Buenos Aires, Ciudad autónoma de Buenos Aires, Argentina; ^2^Department of Immunology, Instituto de Medicina Experimental, Academia Nacional de Medicina (CONICET), Academia Nacional de Medicina de Buenos Aires, Ciudad autónoma de Buenos Aires, Argentina; ^3^Department of Pathology, Instituto de Estudios Oncológicos, Academia Nacional de Medicina de Buenos Aires, Ciudad autónoma de Buenos Aires, Argentina; ^4^Retired, Ciudad autónoma de Buenos Aires, Argentina; ^5^Department of Pathology, University of Washington, Seattle, WA, United States

**Keywords:** murine tumors, antitumor vaccines, immune-checkpoints inhibitors, tumor-immunostimulation, immunosurveillance

## Abstract

Immune-checkpoint inhibitors and antitumor vaccines may produce both tumor-inhibitory and tumor-stimulatory effects on growing tumors depending on the stage of tumor growth at which treatment is initiated. These paradoxical results are not necessarily incompatible with current tumor immunology but they might better be explained assuming the involvement of the phenomenon of tumor immunostimulation. This phenomenon was originally postulated on the basis that the immune response (IR) evoked in Winn tests by strong chemical murine tumors was not linear but biphasic, with strong IR producing inhibition and weak IR inducing stimulation of tumor growth. Herein, we extended those former observations to weak spontaneous murine tumors growing in pre-immunized, immune-competent and immune-depressed mice. Furthermore, we demonstrated that the interaction of specifical T cells and target tumor cells at low stimulatory ratios enhanced the production of chemokines aimed to recruit macrophages at the tumor site, which, upon activation of toll-like receptor 4 and p38 signaling pathways, would recruit and activate more macrophages and other inflammatory cells which would produce growth-stimulating signals leading to an accelerated tumor growth. On this basis, the paradoxical effects achieved by immunological therapies on growing tumors could be explained depending upon where the therapy-induced IR stands on the biphasic IR curve at each stage of tumor growth. At stages where tumor growth was enhanced (medium and large-sized tumors), counteraction of the tumor-immunostimulatory effect with anti-inflammatory strategies or, more efficiently, with selective inhibitors of p38 signaling pathways enabled the otherwise tumor-promoting immunological strategies to produce significant inhibition of tumor growth.

## Introduction

Since the classical work of Prehn and Main (1) demonstrating that vaccination against chemically induced murine tumors was feasible, there were numerous attempts to treat human tumors using immunological strategies. Although most of the former trials were disappointing, a deeper understanding of the cellular and molecular aspects of the immune response (IR), achieved in the last 20 years, prompted the development of new schedules of immunotherapy against cancer, including vaccines combined with anti-Treg or anti-myeloid-derived suppressor cells antibodies and, more recently, immune-checkpoint inhibitors, such as antibodies against cytotoxic T lymphocyte-antigen 4 (CTLA-4) or against programmed death-ligand 1 (PD-L1) (2–6).

**Figure 1:**
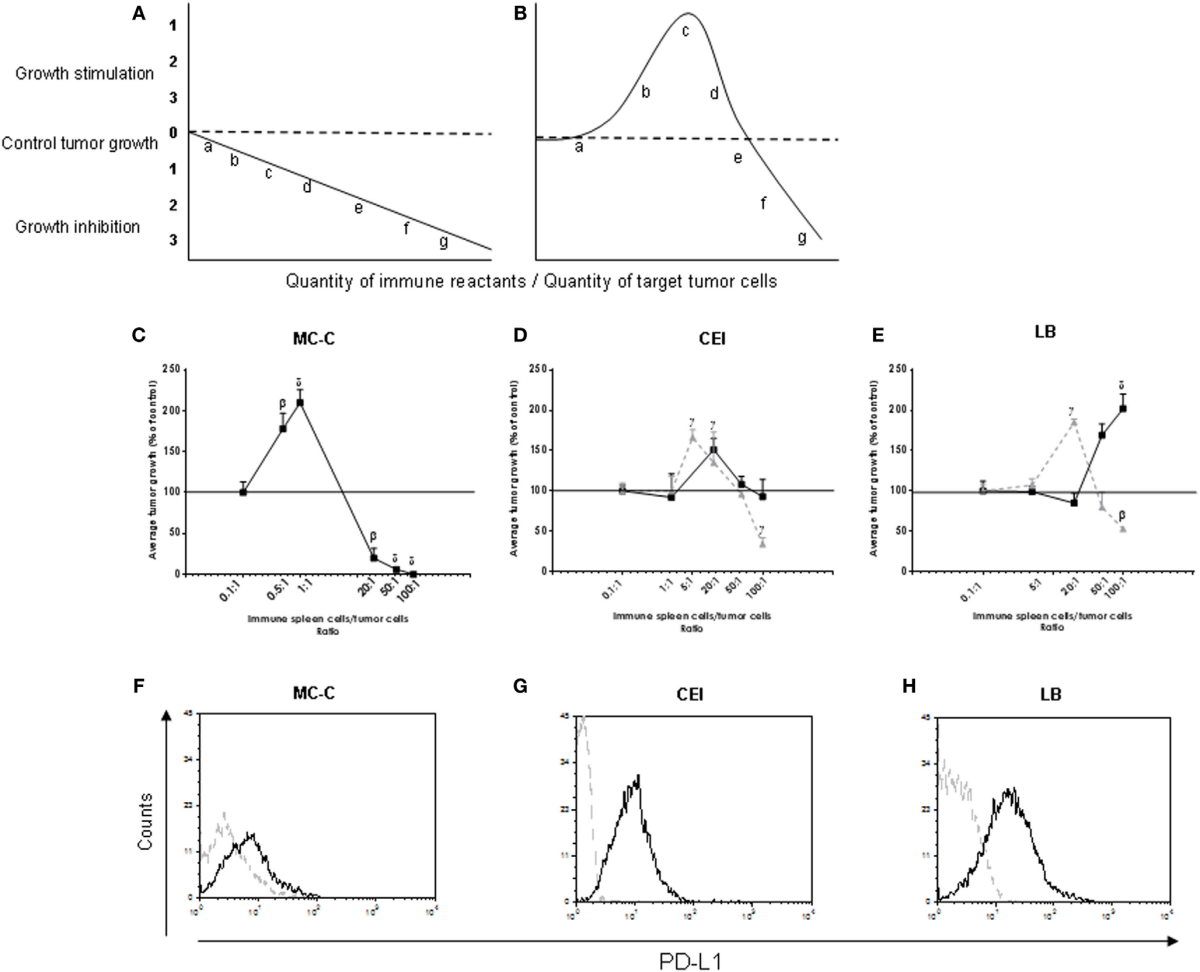
**(A,B)** Idealized linear **(A)** and biphasic **(B)** antitumor immune reaction curve relating the *immune reactants/tumor cells ratio inoculated into test mice* (*x*-axis) with *tumor growth* (*y*-axis). Letters *a, b, c, d, e, f*, and *g* indicate different ratios between immune reactants and target tumor cells. Tumor growth was expressed as a percentage of control tumor growth which was that observed with tumor cells alone and is represented by the horizontal dashed line. **(C–E)** Real biphasic antitumor immune response curve evaluated in Winn tests relating the *immune spleen cells/tumor cells ratio inoculated into euthymic or nude test mice* (*x*-axis) with *tumor growth* (*y*-axis). Data from both euthymic and nude test mice were very similar and, in consequence, were pooled. The tumor cells were obtained from the strongly immunogenic MC-C tumor **(C)**, and from the tumors of undetectable immunity CEI **(D)** and LB **(E)**. Spleen cells were obtained from normal mice or mice immunized against tumor cells (MC-C, CEI, or LB) by pretreatment with X-lethally irradiated homologous tumor cells that had been either untreated *[full line: (*▪*)]* or treated, before being irradiated, with an inhibitor of programmed death-ligand 1 (PD-L1) expression (JQ1, 200 nM in culture for 48 h) *[dashed line: (*▴*)]*. For MC-C, both curves were virtually identical, and for simplicity only the normal line was shown. Tumor growth, initiated in all cases with 1 × 10^5^ tumor cells, was expressed as a percentage of control tumor growth which was that observed with tumor cells alone or mixed with normal spleen cells and is represented by the horizontal line in 100% (for simplicity, SE of control—that was lower than 10% of the mean value, in the three tumor models—was not represented in the Figure). Differences were observed throughout tumor growth. By simplicity, values at day 35 of tumor growth for MC-C and CEI and at day 18 for LB were registered in the Figure taking into account that MC-C and CEI tumors grow significantly lower than LB tumor. It is worth noting that tumor mass at these days predicted the survival time: the larger the tumor mass, the shorter the survival time. The curves represent the mean ± SE of five (for MC-C) and four (for CEI and LB) independent experiments. Each point of each experiment represents the mean of 4–6 mice. Statistics: ^α^*p* < 0.05; ^β^*p* < 0.02; ^γ^*p* < 0.01; ^δ^*p* < 0.001, as compared with control. **(F–H)** Expression of PDL-1. Representative experiment showing the content of surface PDL-1 in MC-C **(F)**, CEI **(G)**, and LB **(H)** tumor cells using flow cytometry.

Presumably, underlying these new schedules, there is the belief that the worst possible outcome for the application of immunological therapies is a null effect on tumor growth because it is assumed that there is a linear and monotonic relationship between the intensity of the antitumor specific IR—resulting from the interplay of positive (for example T cytotoxic cells) and negative (for example Treg cells) immunological forces—and the level of tumor inhibition (Figure 1A).

However, a significant body of evidence (7–9) suggests that the antitumor IR may be not linear but biphasic, with strong IRs inducing inhibition while weak ones inducing stimulation of tumor growth (Figure 1B). The biphasic nature of the IR curve on which the so-called immunostimulatory theory of cancer was based, entails the ambiguous suggestion that immunological strategies might be good therapeutic options against cancer but also might run a real risk of doing harm if the immunity induced by them is too weak to move the reaction beyond the tumor-stimulatory part of the IR curve (8, 10).

Despite its important theoretic and therapeutic potential consequences, the immunostimulatory theory of cancer has usually been neglected on the basis of three arguments.

First, the experiments that ultimately support this theory were performed using strongly immunogenic chemically induced murine tumors (7), which might say little about the evolution of the antitumor immunity during natural carcinogenesis (2, 11–13). Second, up to date, the concept of “tumor immunostimulation” lacks mechanistic support. It is now recognized that some effectors of the chronic non-specific inflammation contribute to tumorigenesis at all stages (8, 13). However, the relationship, if any, between this non-specific tumor-promoting effect and that attributed by the immunostimulatory theory of cancer to a weak specific IR remains to be elucidated.

Third, the immunostimulatory theory of cancer has not been directly tested in the context of immunotherapeutic assays against growing tumors.

In this work, we evaluated whether the phenomenon of tumor-immunostimulation is not only related to strongly immunogenic tumors but also to murine tumors exhibiting weak and undetectable immunogenicity, most of spontaneous origin, which have been considered the best models for most human cancers (11, 14, 15). We also tested other predictions of the immunostimulatory theory of cancer, including therapeutic attempts to treat growing tumors using vaccines, immune-depressors, and immune-checkpoint inhibitors and explored putative mechanisms underlying the phenomenon of tumor immunostimulation.

## Materials and Methods

### Animals

*Euthymic, thymectomized at birth*, and *toll-like receptor 4 (TLR4)-KO* BALB/c mice were raised in our colony. *Nude* BALB/c and *NOD scid gamma (NSG)* mice were purchased from Comisión Nacional de Energía Atómica and Instituto de Biología y Medicina Experimental, Argentina, respectively. Thymectomy in newborn mice, macrophage-depleted, and B-cell-depleted mice were performed as described (16, 17). Care of mice was according to the NIH Guide and Use of Laboratory Animals, and was approved by the Committee for the Care and Use of Laboratory Animals (CICUAL) of our Institution, IMEX-CONICET, Academia Nacional de Medicina de Buenos Aires. Experiments were routinely done on euthymic mice unless otherwise stated.

### Murine Tumors

MC-C:strongly immunogenic fibrosarcoma induced by the chemical 3-methylcholanthrene.
CEI:spontaneous undifferentiated carcinoma exhibiting undetectable immunogenicity.
LB:spontaneous T-lymphoid leukemia-lymphoma exhibiting undetectable immunogenicity.
C7HI:medroxyprogesterone acetate-induced mammary adenocarcinoma exhibiting undetectable immunogenicity.

Twelve additional tumors, mostly of spontaneous origin, that were used in selected experiments, are indicated in Table 1. All tumors were previously described (3, 16–21). Tumor dose 50 (TD_50_): number of tumor cells able to grow in 50% of mice. Tumor volume was calculated as 0.4*ab*^2^, where *a* and *b* are the larger and smaller diameters, respectively (18–20). Medium was RPMI 1640 (Gibco) supplemented as described (3). Tumor lysates, histological, and immunohistochemical analysis were performed as previously reported (3).

**Table 1:** Effect of immunization procedures on the growth of apparently non-immunogenic murine tumors.

Tumors	TD_50_ (mean ± SE)			Method
	Control	Immunized*		
		Conventional (#Experiments)	+JQ1 (#Experiments)	
LB^a^	1,093 ± 33	628 ± 123^γ^ (6)	6,667 ± 883^γ^ (3)	1, 2
C7-HI^b^	5,500 ± 800	6,200 ± 850^NS^ (4)		1, 3
L15-S^a^	1,300 ± 150	500 ± 50^α^ (3)		1
CEI^a^	13,100 ± 2,400	3,150 ± 200^β^ (3)	33,400 ± 6,400^α^ (2)	1, 3
T2280^c^	35,300 ± 2,600	22,050 ± 500^γ^ (3)		3
CM1^a^	23,350 ± 4,650	23,350 ± 4,650^NS^ (3)		1, 3
S-180^a^	22,700 ± 2,300	8,900 ± 1,066^γ^ (3)		3
Fib1^a^	5,600 ± 150	3,200 ± 300^β^ (2)		1
Cspp^a^	32,000 ± 3,000	6,800 ± 500^β^ (2)		3
CPV^a^	3,200 ± 500	3,200 ± 500^NS^ (2)		3
CM2^a^	6,600 ± 250	1,800 ± 150^α^ (2)		3
CM3^a^	30,000 ± 2,500	3,200 ± 1,000^γ^ (2)		3
PS^a^	4,500 ± 100	2,200 ± 100^γ^ (2)		1
P245^a^	4,600 ± 580	1,700 ± 50^α^ (2)		1
Fib2^a^	8,900 ± 400	6,700 ± 400^α^ (2)		1,3
MC-C^d^ (positive control)	48,000 ± 2,600	>5,000,000^δ^ (6)	>5,000,000^δ^ (3)	1–3

*^a^Spontaneous tumor*.

*^b^Induced in a BALB/c female mouse treated with 40 mg of medroxyprogesterone acetate (MPA) every 3 months for 1 year and thereafter maintained as an MPA-independent line*.

*^c^Induced by a novel exogenous MMTV in a BALB/c female mouse*.

*^d^Induced after implantation of a methylcholanthene pellet*.

*LB, L15-S, PS, and P245 are lymphomas. C7-HI, CEI, CM1, CM2, CM3, Cspp, T2280, and CPV are carcinomas. S-180, Fib1, Fib2, and MC-C are fibrosarcomas*.

*TD_50_, number of tumor cells able to grow in 50% of mice; #Experiments, number of experiments for each tumor including control and conventional immunized groups, and control, conventional immunized, and immunized + JQ1 groups. For each tumor and each experiment, 11–18 controls and 11–19 putatively immunized mice were used*.

*Immunized.

*Method: Conventional Immunization procedures*.

*1: pretreatment with two doses of X-lethally irradiated tumor cells 14 and 7 days before tumor challenge*.

*2: pretreatment with two doses of dendritic cells pulsed in vitro with tumor lysates and then inoculated in mice 14 and 7 days before tumor challenge*.

*3: tumor implantation and extirpation 14 days before tumor challenge*.

+JQ1

*Immunized with X-lethally irradiated tumor cells pretreated with an inhibitor of programmed death-ligand 1 expression (JQ1 200 nM for 48 h before being irradiated)*.

*Statistical significance*.

*^α^p < 0.05; ^β^p < 0.02; ^γ^p < 0.01; ^δ^p < 0.001; NS, not significant, as compared with control*.

### Winn Test

Antitumor activity of spleen cells was investigated by mixing them *ex vivo* with tumor target cells at different effector–target ratios. The mixtures were then inoculated by the subcutaneous (s.c.) route into test mice and tumor growth evaluated. The magnitude of tumor inhibition is considered a measure of the antitumor activity of spleen cells (3).

### Antitumor Vaccination Strategies and Other Techniques

Tumor implantation and excision, pretreatment with X-lethally irradiated (LI) tumor cells, and pretreatment with dendritic cells incubated with tumor lysate were carried out as reported (3, 9, 16). Isolation of macrophages, [^3^H]-thymidine uptake assay, and cell-mediated cytotoxicity against ^51^Cr-labeled cells were performed as described (3, 17, 19).

### Drugs, Cytokines, and Chemokines

The T-immune-depressor *cyclosporine A* (Sandoz), the anti-inflammatory *indomethacin* (Sigma-Aldrich), the selective p38 inhibitor *SB202190* (Santa Cruz Biotechnology), and the pro-inflammatory *thyoglycollate* (Britania Laboratory, Argentina) were used as reported (3, 17, 22, 23).

TNF-α, IL-1β, and IL-6 were quantified using ELISA kits from R&D Systems. RANTES and MIP-1α chemokines that control macrophage migration were evaluated using ELISA kits from Pepro-Tech, following manufacturer’s recommendations.

### Immune-Checkpoint Inhibitors

JQ1 (Sigma-Aldrich), an inhibitor of PD-L1 expression was used in culture as described (24). Blocking anti-mouse PD-L1, clone 10F.9G2 and anti-mouse CTLA-4 (CD152), clone 9H10 (BioXCell) were used as described (25).

### Flow Cytometry

Tumor cells were incubated with specific rat anti-mouse PD-L1, clone MIH5 (Ap-Biotech, Argentina) following manufacturer’s recommendations. Fluorescence of individual cells was measured in a flow cytometer (Becton Dickinson) and was analyzed with Cell Quest and ModFit softwares (Becton Dickinson). More details were given elsewhere (3).

### Western Blotting

Western blotting was carried out with standard techniques as described (3) and analyzed by ImageQuant software. Anti-p38 (Santa Cruz Biotechnology) and anti-β actin (Cell Signaling Technology) monoclonal antibodies were used. Levels of p38 were normalized with β actin densitometry units as reported (21).

### Statistical Analysis

Student’s *t*-test was used. Values were expressed as mean ± SE. Differences were considered to be significant whenever *p* value was ≤0.05.

## Results

### The IR Curve Associated with Tumors Displaying Different Degrees of Immunogenicity

The antitumor activity of spleen cells from mice putatively immunized against the strongly immunogenic MC-C (3) or the apparently non-immunogenic CEI or LB tumors (18–21) (using pretreatment with X-lethally irradiated homologous tumor cells as immunization procedures) was evaluated using Winn tests by mixing those spleen cells with the corresponding tumor cells at different spleen cells/tumor cells ratios. Then, the mixtures were inoculated by the s.c. route into euthymic or nude mice and tumor growth was determined. Tumor cells inoculated alone or mixed with normal spleen cells (NSCs) rendered similar results and served as controls. In all cases, the number of tumor cells was the same: 1 × 10^5^. As shown in Figure 1C, the IR curve for MC-C tumor displayed both tumor-inhibitory and tumor-stimulatory effects at high (50/1 or 100/1) and low (0.5/1 or 1/1) *spleen cells/tumor cells* ratios, respectively, and was virtually identical to the idealized biphasic IR curve depicted in Figure 1B. As for CEI and LB tumors, no inhibitory effects were detected at any point of the IR curve, although a significant tumor-stimulation was observed at high ratios (Figures 1D,E). The absence of inhibitory-mediated immune effects against CEI and LB tumors could be associated, at least in part, with the fact that both tumors (different to that occurred with MC-C tumor) displayed high expression of PD-L1 that could prevent the onset of an inhibitory antitumor IR (Figures 1F–H). Supporting this contention, when Winn tests were carried out mixing CEI or LB tumor cells with spleen cells collected from mice immunized with X-lethaly irradiated tumor cells that had been pretreated with an inhibitor of PD-L1 expression (JQ1), a significant inhibition was detected at high ratios while the tumor-stimulating effect moved toward lower ratios. In contrast, pretreatment with JQ1 did not modify the IR curve for MC-C tumor that displayed low content of PD-L1.

When Winn tests were performed using spleen cells immune to one tumor mixed with cells from another tumor, or spleen cells from putatively immunized nude mice, neither tumor-inhibitory nor tumor-stimulatory effects were observed, suggesting that both were specific and T-cell dependent.

### Effect of Pre-immunization on the Growth of Apparently Non-immunogenic Tumors

The growth of 15 murine tumors, mostly of spontaneous origin, was analyzed in both untreated and putatively immunized mice. As shown in Table 1, in not a single case the conventional immunization procedures produced an inhibitory effect on tumor growth; actually, 12 out of 15 tumors were stimulated in the “immunized” mice. Similar to that occurred with Winn tests, the tumor-stimulatory effect induced by pre-immunization was specific and T dependent.

This suggested that the 12 stimulated tumors bore weak antigens that only seemed to induce stimulatory IRs. However, when immunization was combined with a conspicuous inhibition of PD-L1 expression by immunizing mice with LI tumor cells that had previously been pretreated with JQ1, the TD_50_ of two out of two spontaneous tumors assayed increased significantly suggesting that upon inhibition of PD-L1 the antigens of these tumors were also capable to induce a tumor-inhibitory IR.

As positive control of immunogenicity, Table 1 also shows that growth of the methylcholanthrene-induced MC-C tumor was strongly prevented in pre-immunized mice.

### Tumor Growth in Mice Displaying Different Degrees of Immune Competence

Growth of MC-C, LB, CEI, and C7HI tumors was evaluated in *euthymic, thymectomized at birth, nude*, and *NSG* mice which exhibited high, medium, low, and undetectable immune competence, respectively. Immune competence was determined by the capacity of spleen cells to kill ^51^Cr-labeled tumor or normal allogeneic cells (not shown).

**Figure 2:**
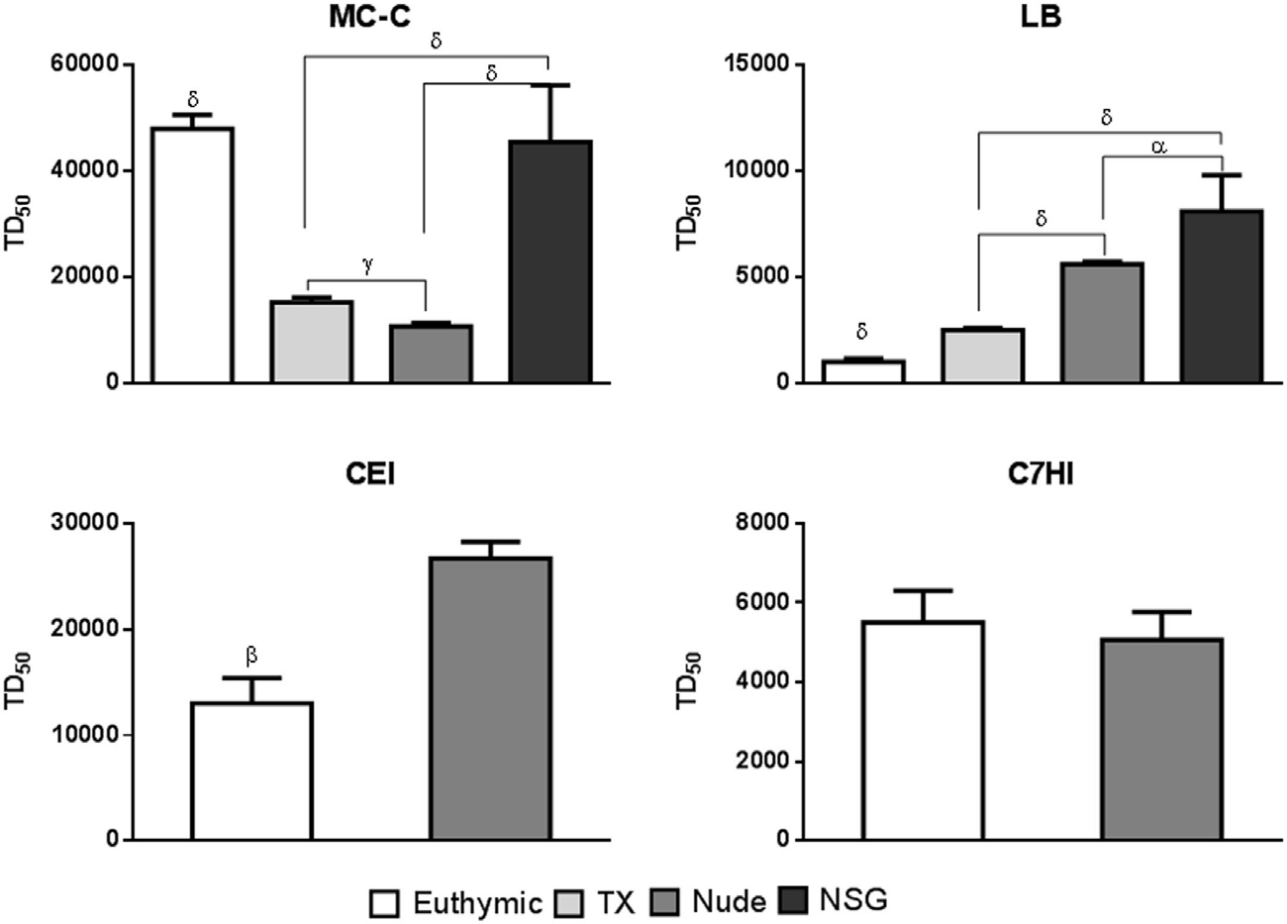
Evaluation of tumor growth in mice displaying different degrees of immune competence. Different strains of mice were used: euthymic, thymectomized at birth (Tx), nude, and Nod Scid Gamma (NSG) that were challenged with different doses of tumor cells to assesses the tumor dose 50 (TD_50_). The tumors used were: MC-C, LB, CEI, and C7HI. MC-C: mean ± SE of six experiments for *euthymic, Tx*, and *nude* mice and mean ± SE of two experiments for *NSG* mice; LB: mean ± SE of six experiments for *euthymic, Tx*, and *nude* mice and mean ± SE of two experiments for *NSG* mice; CEI: mean ± SE of three experiments for *euthymic* and *nude* mice; C7HI: mean ± SE of four experiments for *euthymic* and *nude* mice. In each experiment, 12–16 mice were utilized. Statistics: ^α^*p* < 0.05, ^γ^*p* < 0.01, ^δ^*p* < 0.001.

As shown in Figure 2, growth of the strongly immunogenic MC-C tumor that was strikingly inhibited by conventional pre-immunization was *inversely proportional* to the immune competence of the host although this relationship did not include *NSG* mice. In effect, in these extremely immune-depressed mice, growth of MC-C tumor was, surprisingly, similar to that attained in euthymic mice.

Exactly opposite results were obtained with the weakly antigenic CEI and LB tumors that were stimulated upon conventional pre-immunization. In effect, growth of both tumors was *directly proportional* to the immune competence of the host.

Finally, growth of C7HI tumor that exhibited undetectable antigenicity (it was neither inhibited nor stimulated upon conventional pre-immunization) was *similar independently of the immune competence of the host*.

### Mechanisms Underlying Tumor-Immunostimulation

**Figure 3:**
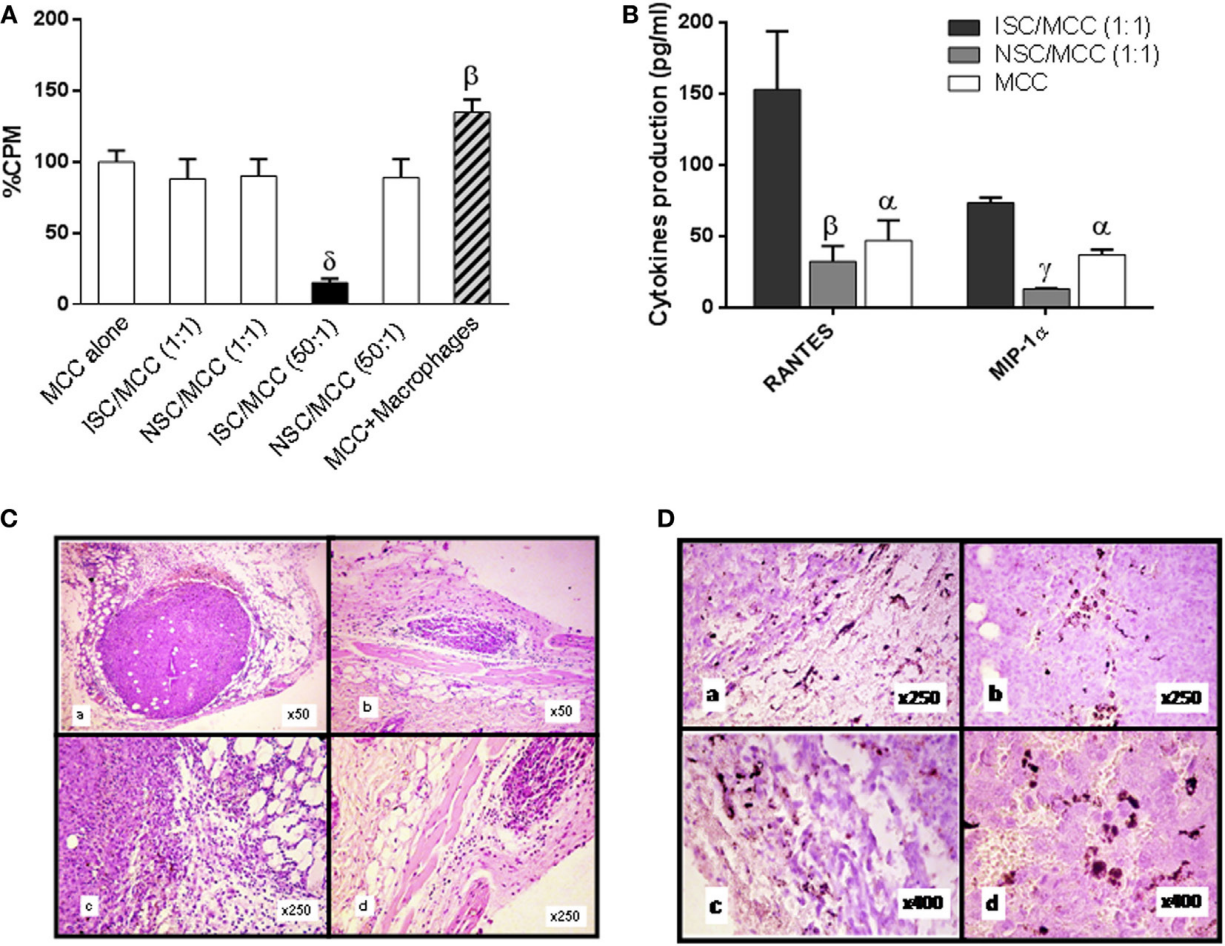
Role of inflammatory components in the immune-mediated tumor-stimulatory effect. **(A)** *In vitro* proliferation of 1 × 10^5^ MC-C tumor cells alone (MCC alone), or admixed with anti-MC-C immune spleen cells (ISC) [obtained from mice immunized against MC-C by pretreatment with X-lethally irradiated MC-C tumor cells] or with normal spleen cells (NSC) at 1:1 or 50:1 ISC/MCC and NSC/MCC ratios or with inflammatory macrophages at 1:1 ratio, as evaluated by using a 18 h ^3^[H]-thymidine uptake assay. Macrophages were collected from the peritoneum of mice that had received 1 ml of 3% of the pro-inflammatory thyoglycollate by the i.p. route, 3 days earlier. The overall ^3^H-thymidine uptake by attached cells was attributed to MC-C tumor cells since ^3^[H]-uptake by macrophages alone was negligible. Number of determinations per group, *n* = 6. Statistics: ^β^*p* < 0.02 as compared with MCC alone, ISC/MCC (1:1), NSC/MCC (1:1), and NSC/MCC (50:1); ^δ^*p* < 0.001 as compared with the other groups. **(B)** Expression of RANTES and MIP-α. Increase in the 24h-conditioned medium of the mixture ISC/MC-C cultured at 1:1 ratio as compared with that of NSC/MCC (1:1) and MC-C alone. The conditioned medium was obtained after culturing 24 h 5 × 10^6^ MC-C tumor cells alone or mixed with 5 × 10^6^ ISC or NSC. The number of determinations was six for RANTES and three for MIP-α. Statistics: ^α^*p* < 0.05; ^β^*p* < 0.02; ^γ^*p* < 0.01 as compared with ISC/MC-C (1:1). **(C)** Growth of MC-C tumor, 8 days after the s.c. implantation of 1 × 10^5^ MC-C tumor cells admixed with 1 × 10^5^ anti-MC-C ISCs (stimulatory mixture) (a,c) or with 1 × 10^5^ NSCs (control) (b,d). **(D)** Immunohistochemistry of MC-C tumor. Increase of CD11b+/F4/80+ (a,c) and CD3+ (b,d), 8 days after the s.c. implantation of stimulatory mixture. Up to day 8, lymphocytes were mainly CD3+ T cells but afterward, CD20+ B220+ B cells were also observed (not shown).

*In vitro* proliferation of MC-C tumor cells mixed with immune spleen cells (ISCs) at different spleen cell/tumor cell ratios is shown in Figure 3A. A striking inhibitory effect was seen at 50/1 ratio, reproducing the *in vivo* observations. In contrast, no stimulatory effect was detected at 1/1 ratio, indicating that the mere interplay between ISCs and tumor cells was not enough to produce the tumor-immune-stimulatory effect.

Several observations suggested that inflammatory components could play a role in the tumor-immunostimulatory effect observed *in vivo*.

First, *in vitro* proliferation of tumor cells was enhanced when they were admixed with non-specifically induced inflammatory macrophages (Figure 3A).

Second, the 24 h-conditioned medium of the stimulatory mixture (*immune spleen cells/tumor cells* at 1/1 ratio) displayed high concentration of chemokines (RANTES and MIP-1α) aimed to induce macrophage migration (Figure 3B).

Third, a more intense tumor-inflammatory infiltration composed by CD11b+ F4/80+ macrophages and, at lesser degree, by lymphocytes, were observed at the place where the stimulatory mixture had been implanted (Figures 3C,D, panel a,c) as compared with that of tumor cells implanted alone or mixed with NSCs (Figures 3C,D, panel b,d).

**Figure 4:**
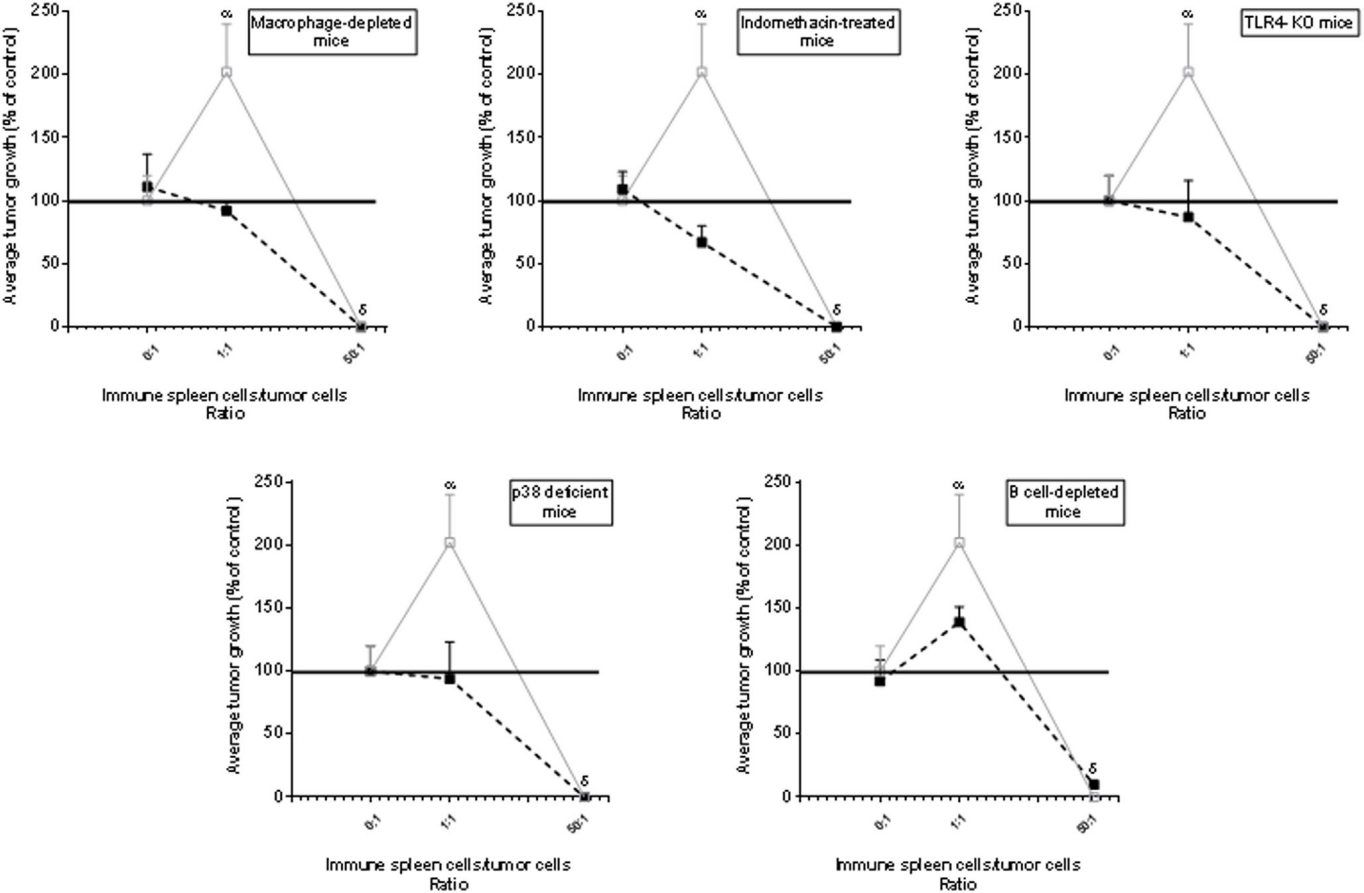
Low or undetectable immune-mediated tumor-stimulatory effect in mice displaying low inflammatory responses. Evaluation of the immune response curve using Winn test in macrophage-depleted mice, indomethacin-treated mice, toll-like receptor 4 (TLR4)-KO mice, p38 locally deficient mice or B-cell-depleted mice (▪). For comparative purposes, we have, in each figure, added the control group carried out using recipient euthymic or nude mice (▫) [data were very similar and for simplicity, were pooled]. *The dose of indomethacin* (0.5 mg/kg) was diluted in 0.015 M NaCl and was inoculated in the i.p. route 1 day before tumor inoculation. The selective p38 inhibitor *SB202190* was inoculated four consecutive days (30 μg/kg/day) at the site of tumor implantation, starting at the day of tumor inoculation. Normal spleen cells (NSCs) were obtained from normal mice and immune spleen cells were obtained from mice immunized against MC-C by pretreatment with X-lethally irradiated MC-C tumor cells. Tumor growth, initiated in all cases with 1 × 10^5^ tumor cells, was expressed as a percentage of control tumor growth which was that observed in euthymic and nude test mice that received tumor cells alone or mixed with NSCs and is represented by the horizontal line in 100% (for simplicity, SE of control—that was lower than 10% of mean value—was not represented in the Figure). Differences were observed throughout tumor growth. By simplicity, values at day 35 of tumor growth were registered in the Figure. Each point of each experiment represents the mean of 4–6 mice. The curves represent the mean ± SE of three independent experiments. Statistics: ^α^*p* < 0.05 as compared with control and 1/1 ratio; ^δ^*p* < 0.001 as compared with control.

Fourth, when Winn tests were carried out in macrophage-depleted, indomethacin-treated, TLR4-KO or p-38-deficient test mice, the stimulatory effect was not observed. In B-cell-depleted mice, a stimulatory effect was detected but it was significantly lower than that observed in euthymic and nude test mice (Figure 4) suggesting that apart from macrophages, B cells could also participate—although at lesser degree than macrophages—in the phenomenon of tumor immunostimulation. In contrast, the inhibitory effect was observed indistinctly in all mice, reinforcing the notion that it was independent on inflammatory mediators. The rationale for using TLR4-KO- and p-38-deficient mice was based on the claim that agonists of TLR4 and activation of p38 MAPK-signaling pathway can be important for the induction of the inflammation associated with neoplastic processes (26, 27).

**Figure 5:**
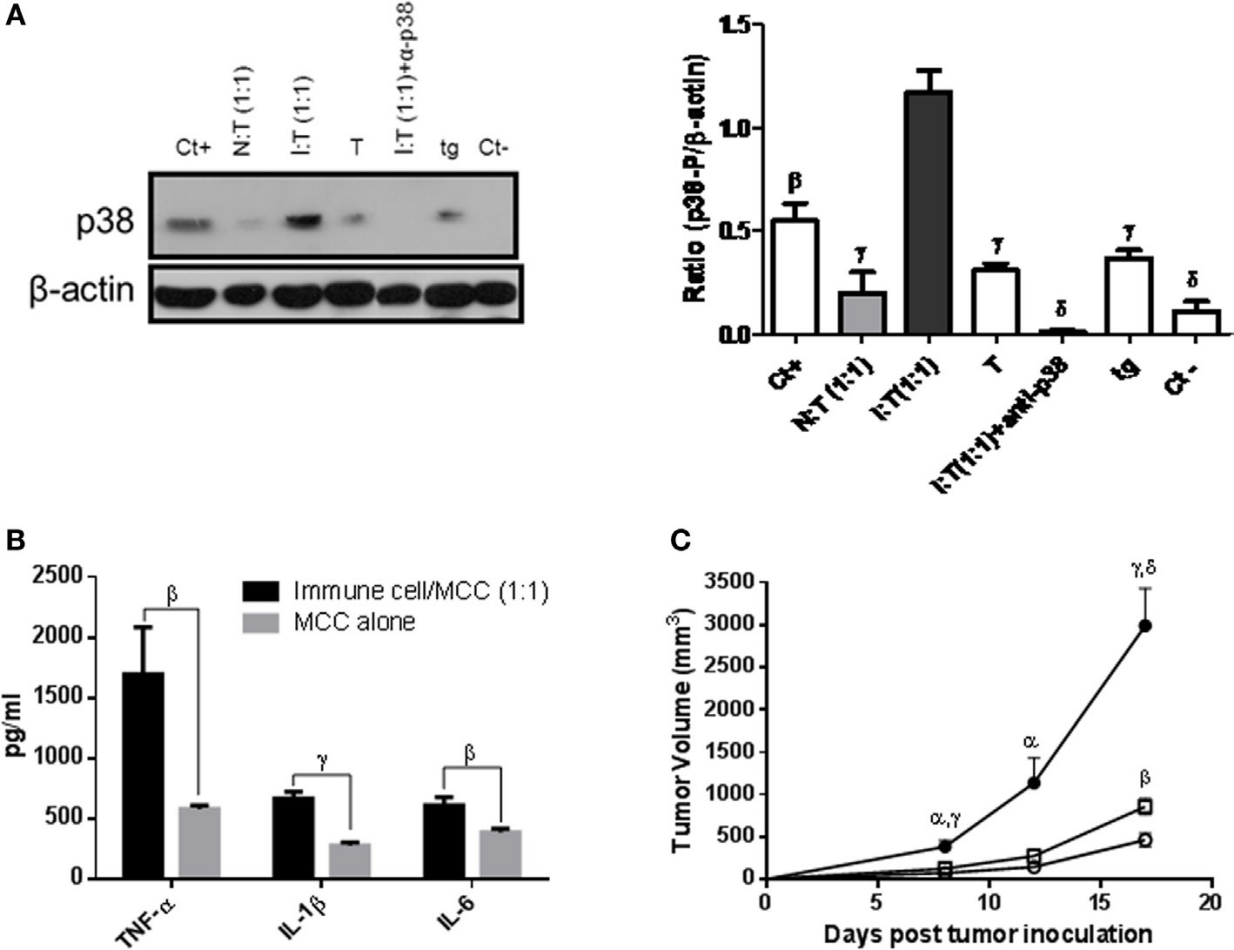
**(A)** Expression of phosphorylated (p)-38 (p38) by Western blotting. Macrophages (3 × 10^6^ cells) collected surrounding the s.c. tumor place (one experiment) or from the peritoneum (two experiments), 3 days after s.c. or i.p. implantation of 1 × 10^5^ MC-C tumor cells alone (T), or mixed with normal spleen cells (NSCs) at 1/1 ratio [N:T (1:1)], with immune spleen cells (ISCs) at 1/1 ratio [I:T (1:1)] or with ISCs at 1/1 ratio plus the p38 inhibitor (days 0–3, 30 μg/kg/day) at the place of tumor inoculum [I:T (1:1) + α-p38]. Intraperitoneal macrophages from normal mice were used as negative controls of p38 expression (Ct−). Pervanadate (OVAN) was used as positive phosphorylation controls (Ct+). Macrophages collected from mice that received 1 ml of 3% thyoglycollate (tg) by the i.p. route, 3 days earlier, served as control of non-specific inflammation. At the left, a representative experiment is shown. At the right, histograms show levels of p38 in the different groups, normalized with beta-actin densitometric units, representing the mean ± SE of three independent experiments. NSCs were obtained from normal mice and ISCs were obtained from mice immunized against MC-C tumor by pretreatment with X-lethally irradiated MC-C tumor cells. Statistics: ^β^*p* < 0.02; ^γ^*p* < 0.01; ^δ^*p* < 0.001 as compared with the I:T (1:1) group. **(B)** Expression of TNF-α, IL-1β, and IL-6 pro-inflammatory cytokines in the 24-h conditioned medium of macrophages collected from the peritoneum of mice that had received, 3 days before, an i.p. inoculation of 1 × 10^5^ MC-C tumor cells alone or mixed with stimulatory mixture at 1/1 ratio [I:T (1:1)]. Statistics: control versus I:T (1:1); ^β^*p* < 0.02; ^γ^*p* < 0.01; mean ± SE of three experiments. **(C)** Effect of macrophages activated by the stimulatory mixture on the growth of MC-C tumor. Euthymic test mice received by the s.c. route 1 × 10^5^ MC-C tumor cells mixed with peritoneal macrophages (1 × 10^5^) of mice that had received i.p. 3 days earlier the *stimulatory mixture* [at 1/1 ratio, (●), *n* = 6]. As a control of non-specific inflammation, MC-C tumor cells were mixed with peritoneal macrophages of mice that received 3% thyoglycollate (tg) [(▫); *n* = 4, i.p. 3 days earlier]. The control groups (○) were, mice that received MC-C tumor cells alone (1 × 10^5^, *n* = 4), or mixed with macrophages collected from mice that had received the normal mixture (at 1/1 ratio, *n* = 4), or none (*n* = 4). The data of the control groups were almost identical and were pooled. Tumor growth was registered in all groups. Statistics: Stimulatory mixture group (●) versus Control groups (○): ^α^*p* < 0.05; ^γ^*p* < 0.01; and ^δ^*p* < 0.001; Stimulatory mixture group (●) versus tg group (▫): ^α^*p* < 0.05 and ^γ^*p* < 0.01 and tg group (▫) versus control groups (○): ^β^*p* < 0.02.

Fifth, the expression of p38 was significantly higher in macrophages collected surrounding the s.c. tumor place in mice receiving the stimulatory mixture than in controls, receiving tumor cells alone or mixed with NSCs. A similar higher expression of p38 was detected in macrophages collected from the peritoneum of mice that had received the stimulatory mixture by the i.p. route as compared with peritoneal macrophages from mice that had received tumor cells alone or mixed with NSCs (Figure 5A). This was correlated with a higher production of pro-inflammatory TNF-α, IL-1β, and IL-6 cytokines (Figure 5B) and a stronger stimulatory effect on tumor growth achieved by these activated macrophages as compared with controls (Figure 5C).

### Therapeutic Antitumor Immunological Schedules: Benefits and Risks

Different antitumor immunological schedules were attempted therapeutically against two murine tumors that had already started their growth, the strongly immunogenic MC-C (low PD-L1 content) and the weakly antigenic LB (high PD-L1 content).

#### Vaccines and Immune-Depressors

**Figure 6:**
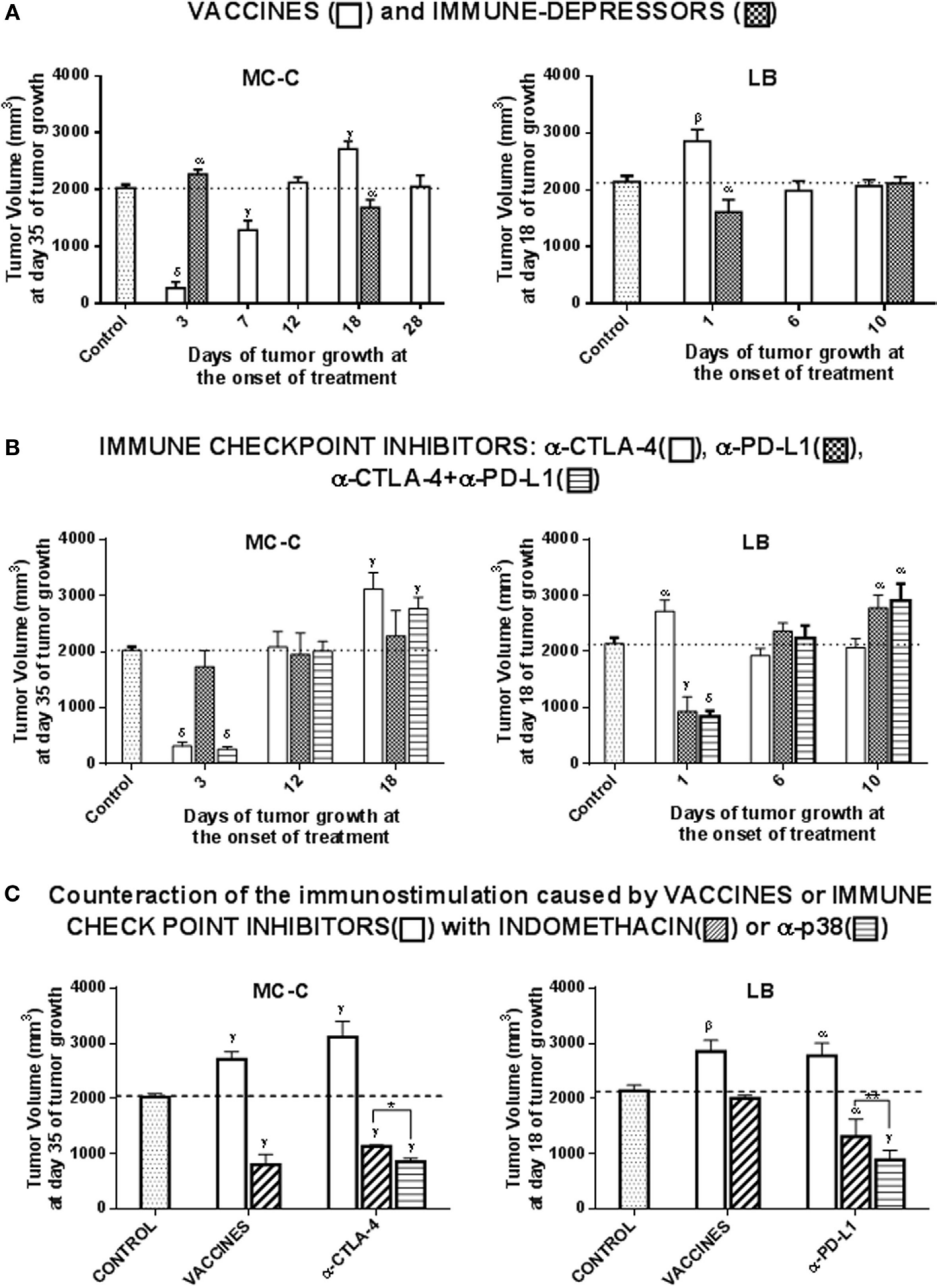
Therapeutic antitumor immunological schedules. **(A)** *Vaccines and Immune-depressors*. Tumor growth was initiated at day 0 with a s.c. inoculum of 1 × 10^5^ MC-C or LB tumor cells in the right flank. Afterward, at selected times after tumor inoculation, different groups of mice received in the left flank an antitumor vaccine [4 × 10^6^ X-lethally irradiated tumor cells or 3 × 10^5^ dendritic cells loaded with tumor lysate (both vaccines rendered similar results and in consequence their data were pooled)] or the immune-depressor Cyclosporine A [inoculated i.p. every day (15 mg/kg/day) starting at day 3 or 18 of MC-C tumor growth or at day 1 or 10 of LB tumor growth]. The group of mice that did not receive any treatment served as control. The Figure shows representative experiments (one for MC-C and one for LB) out of four experiments for MC-C and three experiments for LB that rendered similar results. Six mice per group were utilized in the representative experiments and data were expressed as mean (mm^3^) ± SE of tumor volume. Statistics: Differences versus control group: ^α^*p* < 0.05; ^β^*p* < 0.02; ^γ^*p* < 0.01; ^δ^*p* < 0.001. **(B)** *Immune-checkpoint inhibitors*. Tumor growth was initiated at day 0 with a s.c. inoculum of 1 × 10^5^ MC-C or LB tumor cells in the right flank. Afterward, different groups of mice received blocking anti-cytotoxic T lymphocyte-antigen 4 (CTLA-4) (inoculated i.p. three times a week), blocking anti-programmed death-ligand 1 (PD-L1) (inoculated i.p. for 13 consecutive days) or both blocking anti-CTLA-4 plus blocking anti-PD-L, starting at selected times (indicated in the Figure) after tumor inoculation. The group of tumor-bearing mice that did not receive any treatment served as control. The Figure shows representative experiments (one for MC-C and one for LB) out of two experiments for MC-C and three experiments for LB that rendered similar results. Four to six mice per group were utilized in the representative experiments and data were expressed as mean (mm^3^) ± SE of tumor volume. Statistics: Differences versus control group: ^α^*p* < 0.05; ^γ^*p* < 0.01; ^δ^*p* < 0.001. **(C)** *Counteraction of the tumor-immunostimulatory effects observed on well-established tumors* (well-established meaning solid tumors that are ≥400 mm^3^). MC-C tumor: tumor growth was initiated at day 0 with a s.c. inoculum of 1 × 10^5^ MC-C tumor cells in the right flank. At day 18 of tumor growth, different groups of mice received an antitumor vaccine alone (X-irradiated tumor cells or dendritic cells loaded with tumor lysate), an antitumor vaccine plus indomethacin, anti-CTLA-4 alone, anti-CTLA-4 plus indomethacin, or anti-CTLA-4 plus SB202190. Anti-CTLA-4 was inoculated i.p. three times a week starting at day 18 of tumor growth. Indomethacin (0.5 mg/kg) was inoculated i.p. once at day 17. SB202190 (30 μg/kg/day) was inoculated i.p. for four consecutive days starting at day 18 of tumor growth. The group of tumor-bearing mice that did not receive any treatment served as control. LB tumor: tumor growth was initiated at day 0 with a s.c. inoculum of 1 × 10^5^ LB tumor cells in the right flank. At day 1 of tumor growth, two groups of mice received an antitumor vaccine alone (X-irradiated tumor cells or dendritic cells loaded with tumor lysate), or the antitumor vaccine plus indomethacin (inoculated i.p. at day 0). At day 10 of tumor growth other groups of mice received anti-PD-L1 alone (inoculated i.p. for 13 consecutive days starting at day 10 of tumor growth), or in combination with indomethacin (inoculated i.p. at day 9 of tumor growth) or with SB202190 (inoculated i.p. for four consecutive days starting at day 10 of tumor growth). The group of tumor-bearing mice that did not receive any treatment served as control. The Figure shows representative experiments (one for MC-C and one for LB) out of three experiments for MC-C and three experiments for LB that rendered similar results. Four to six mice per group were utilized in the representative experiments and data were expressed as mean (mm^3^) ± SE of tumor volume. Statistics: differences versus control group: ^α^*p* < 0.05; ^β^*p* < 0.02; ^γ^*p* < 0.01; ^δ^*p* < 0.001. Difference between anti-CTLA-4 plus indomethacin group versus anti-CTLA-4 plus SB202190 group: *p < 0.02. Difference between anti-PD-L1 plus indomethacin group versus anti-PD-L1 plus SB202190 group: **p < 0.05. *y*-axis: tumor volume (mm^3^). **(A,B)** *x*-axis: days of tumor growth at the onset of treatment. For MC-C tumor, days 3, 7, 12, 18, and 28 of tumor growth corresponded to tumor volumes of <10, 50, 250, 600, and 1,500 mm^3^, respectively. For LB tumor, days 1, 6, and 10 of tumor growth corresponded to tumor volumes of <10, 300, and 700 mm^3^, respectively. **(C)** *x*-axis: treatments. Differences were observed throughout tumor growth. By simplicity, values at day 35 of MC-C tumor growth and at day 18 of LB tumor growth were registered in the figures taking into account that MC-C tumor grows significantly slower than LB.

Antitumor vaccines inhibited MC-C tumor growth when they faced incipient tumors. Afterward, the vaccines produced null or stimulatory effects on tumor growth (Figure 6A).

In contrast, the vaccines induced an accelerated LB tumor growth when they faced incipient tumors. Afterward, neither inhibitory nor stimulatory effects were observed.

In both tumor models, the immune-depressor cyclosporine A produced opposite effects to those observed with the vaccines, although both tumor-inhibitory and tumor-stimulatory effects were rather modest.

#### Immune-Checkpoint Inhibitors

A conspicuous inhibition on MC-C tumor was observed when an anti-CTLA-4 treatment was initiated at early stages of tumor growth; however, as tumor grew, tumor-stimulatory effects were observed (Figure 6B). On the other hand, no effect was achieved at any stage of MC-C tumor growth when anti-PDL-1 antibodies were used.

In contrast, an accelerated growth of LB tumor was observed when an anti-CTLA-4 treatment was initiated at early stages of tumor growth; afterward, no effect was detected. On the other hand, a conspicuous inhibition of LB tumor was achieved with anti-PDL1 antibodies although it was restricted to incipient tumors only.

The administration of anti-CTLA-4 together with anti-PD-L1 antibodies slightly improved the antitumor effects of each separately, but, again, only when small tumors were concerned.

#### Counteraction of the Tumor-Immunostimulatory Effects

The administration of indomethacin or, more efficiently, SB 202190 (selective p38-inhibitor), counteracted the tumor-stimulatory effects achieved by antitumor vaccines or immune-checkpoint inhibitors and produced a significant inhibition of medium- and large-sized established tumors in both the strongly immunogenic and the weakly antigenic models (Figure 6C). Indomethacin or SB202190 alone did not produce any effect.

## Discussion

Immunological strategies have been claimed to be promissory for the treatment of cancer because they could, at least theoretically, circumvent the limitations of conventional non-specific antitumor therapies (2).

However, to date, most attempts to cause an immunologically mediated regression of animal and human tumors, once well-established (28) have not been very successful (3, 8, 28, 29). This has usually been attributed to the putative weak antigenicity of spontaneous tumors (11, 15, 16, 28) and/or to the emergence of tumor- associated negative immune-regulatory mechanisms (3, 30, 31). Another alternative, suggested by the immunostimulatory theory of cancer, postulates that current immunological therapies directed against naturally arisen tumors usually produce weak antitumor IRs that would promote rather than inhibit tumor growth (7, 10) which, in turn, was inspired by the possibility that a weak IR to a fetus (32) may be beneficial for fetal survival. However, the immunostimulatory theory of cancer and many experiments that seemed to support it (7, 8), were developed when our understanding of the immune system was limited, and up to date, the mechanisms underlying that tumor-stimulatory effect have not yet been elucidated (8).

In this work, we have extended the empirical basis of the immunostimulatory theory of cancer suggesting that the IR curve evoked by most murine tumors is not linear but biphasic. This contention was supported by classical immunological assays and by the demonstration that many spontaneous murine tumors, formerly claimed to be non-antigenic (11), grow faster in pre-immunized hosts and more slowly when transplanted in immunocompromised mice. To explain these observations, we only must assume that many “non-immunogenic” tumors are in fact weakly antigenic when transplanted in conventional mice, and generate a stimulatory IR that is placed to the left in the biphasic IR curve (see Figure 1B), for example in “b.” In that case, any conventional vaccination, aimed to enhance that antitumor IR could move the reaction toward “c,” producing accelerated tumor growth although more stringent strategies such as immune-checkpoint inhibitors could, upon certain circumstances, move the IR up to the inhibitory zone of the IR curve. Others have already observed similar tumor-enhancing effects after conventional pre-immunization against spontaneous murine tumors (11, 33). Reciprocally, when they were transplanted in immunocompromised mice, the IR could be moved toward “a,” where tumor growth would be retarded. The relatively few cases of spontaneous tumors that were neither inhibited nor stimulated by pre-immunization and/or immunodepression can also be explained assuming that the IR evoked by them is placed between “0” and “a” where no perceptible effects by altering the immunological state of the host could be anticipated.

In contrast, the strongly immunogenic methylcholanthrene-induced murine tumor utilized in this work, as well as other strongly immunogenic murine tumors reported in the literature (3, 7, 20, 29), were inhibited by pre-immunization and grew faster in immune-depressed mice. This behavior can be explained assuming that the IR mounted against strongly immunogenic tumors inoculated in conventional mice, is probably placed to the right in the IR curve, for example near “f.” In that case, a preventive vaccination will move the reaction toward “g” (to the right), which would produce stronger tumor inhibition. Reciprocally, in immunocompromised mice, the IR will be moved to the left, toward “e” or “d,” producing faster tumor growth. However, when the immune competence of mice was extremely low, as occurred in *NSG* mice (34), tumor growth returned to values observed in euthymic mice which can be elucidated assuming that, in that case, the IR moved far to the left surpassing the curve hump toward “a.”

The above considerations can help overcome a major obstacle for the acceptation of the immunostimulatory theory of cancer namely the fact that it relativizes the currently accepted premise that states that, in the natural history of *all* cancers, the earliest antitumor IR is *inhibitory*. This claim is based on the fact that the overall incidence of cancer is augmented in immunosuppressed individuals. However, this overall incidence is supported by a striking increased incidence of only a few tumor types, while the incidence of most other tumors is marginally augmented, not augmented or actually lowered upon immune-depression, in both animals and men (8, 12, 13, 35–39). Some authors have questioned this conclusion showing that some new developed molecularly defined murine models of immunodeficiency such as GM-CSF/IFN-γ−/− doubly deficient and GM-CSF/IL-3/IFN-γ−/− triply deficient mice, display a higher incidence of many tumor types apparently supporting the immunosurveillance predictions. However, these models are also highly susceptible to bacterial infection displaying acute and chronic inflammation in many organs. The fact that tumor development can be prevented by maintaining the mice on broad-spectrum antibiotics from birth, suggests that the state of chronic inflammation induced by the infections, rather than a putative depression of specific antitumor IRs would be the main condition for cancer development (37). Further, *NSG*, that is one of the most immune-deficient strain of inbred laboratory mice described to date display lower—instead higher—incidence of spontaneous tumors than other less immune-deficient mice (40).

The contention that the incidence of most tumors is not significantly augmented and, in fact, in some cases, is lowered by immune-depression might be explained assuming that, in most cases, the earliest IR evoked during the natural history of carcinogenesis is similar to that observed during the experimental transplantation of spontaneous murine tumors, that is, it is placed to the left of the biphasic IR curve, between “0” and “a” or between “a” and “c.” For example, the much more frequent lymphoglandular complexes associated with the rectum, in comparison to that associated with elsewhere in the colon, led Stewart to suggest that immunostimulation of cancers may be greater in the rectum than in the rest of the bowel and that the lower incidence of rectal malignancies in immunodepressed patients may, therefore, be the result of the loss of much of this normal tumor-stimulation (38).

Furthermore, the theory might also account even for the cases in which immunodepression strikingly enhance the incidence of highly immunogenic methylcholanthene-induced sarcomas in the mouse or Kaposi’s sarcoma and skin and hematological malignancies in the man. At first sight, the behavior of these tumors seems to be only compatible with the immunosurveillance theory. However, can one really be sure that an immunodepression diminishes an initial inhibitory response rather than augments a stimulatory one? If the latter possibility were true, the earliest IR directed to these nascent tumors might also be considered as stimulatory. Two lines of observations support this contention. First, methylcholanthrene-induced sarcomas are usually strongly immunogenic when tested in syngeneic mice, but fail to show immunogenicity in the very mouse in which the tumor had originated, unless the animal is hyper-immunized (41–43); instead, they seem to be stimulated (41, 44). If even these tumors, paradigms of strong immunogenicity, are immune-stimulated rather than immune-inhibited when growing in their autochthonous hosts, it seems to us unlikely that in the early evolution of the same tumors, the incipient and, therefore, weaker antitumor IR could be inhibitory. In the second place, Kaposi’s sarcoma, a prototypical lesion in AIDS patients, commonly “flares” during the period of immune recovery while the AIDS is being treated. This suggests that the tumor grows best when the immune capacity of the patients is still impaired, but not too impaired (45).

The immunoediting hypothesis (13) extends the immunosurveillance theory suggesting that the immune system not only controls the origin of tumors but also sculpts their antigenic profiles, by *negatively* selecting tumor cells that are poorly or non-antigenic or that are able to subvert the IR of the host. However, if the early IR were not inhibitory but stimulatory, clones with low antigenicity, displaying the ability to induce a tumor-stimulating IR would be *positively* selected. In some cases (46), this reaction could continue to stimulate the growth of such tumors long after their inception. In other cases, the initial weak stimulatory response could, as it develops, become inhibitory. In these cases, the initial “*S*” (stimulation) of the immunostimulatory theory of cancer should, as it was previously suggested (47), be integrated with the three “*Es*” (elimination, equilibrium, escape) of the immunoediting one.

Another obstacle for the acceptation of the immunostimulatory theory of cancer has been its lack of mechanistic support. In this work we suggested that the interaction between immune spleen T cells with tumor cells at a low ratio (mimicking a weak antitumor-IR), would produce a significantly higher concentration of chemokines (such as RANTES and MIP-1α) aimed to attract macrophages at the tumor site, than that produced by tumor cells alone or mixed with NSCs. In turn, these macrophages, upon activation of TLR4 and p38 signaling pathways would release pro-inflammatory mediators that would recruit and activate more macrophages and other inflammatory cells—for example B cells, as our experiments and a previous work (47) have suggested—at the tumor site that might produce tumor-growth-stimulating signals leading to an accelerated tumor growth. A rather similar mechanism has recently been proposed to explain how a T-cell dependent adaptive IR can promote the progression of pre-neoplasia to cancer in an established mouse model of prostate cancer (48). In fact, the stages of solid tumors in which tumor cells begin their exponential growth and the antitumor IR is significantly *impaired* or *weakened* by not yet fully understood tumor-associated mechanisms (3, 30), are characterized by a chronic inflammatory condition with *moderately* elevated levels of NF-κB activity in both tumor and inflammatory cells. The notion that such *moderate* and constitutive activity of NF-κB exerts a *pro-tumorigenic* effect is suggested by the fact that patients with chronic inflammatory diseases have significantly higher risks for cancer than the general population (49). In contrast, acute inflammatory processes involving *full* activation of NF-κB, produce *antitumor* effects since are usually accompanied by a *high* activity of cytotoxic immune cells against cancer cells (49).

The arguments presented herein have not merely theoretic value but also practical consequences, since they could explain the paradoxical effects of different immunological strategies on the treatment of established tumors in both experimental (9, 10) and clinical settings (47). In effect, these paradoxical effects might be understood depending upon where the system stands on the biphasic IR curve at each stage of tumor growth, which in turn, would depend upon the antigenic profile of the tumor, the immunogenic strength of the immunotherapeutic schedule, and the immunological state of the host (3, 9).

In our experiments, both antitumor vaccines and immune-checkpoint inhibitors were effective to restrain tumor growth, although only when they faced incipient or small-sized tumors. Afterward, as tumor became larger, and the IR was expected to be moved to the left in the IR curve—because during tumor progression tumor cells usually grow faster than immune reactants (9)—null and stimulatory effects on tumor growth were observed.

At the time when immunostimulation was evident, some form of mitigation of immunity was therapeutically advantageous although the beneficial effects were rather modest. In our hands, the most significant inhibition of medium- and large-sized chemically induced and spontaneous tumors was accomplished by combining vaccines or immune-checkpoint inhibitors with strategies aimed to counteract tumor immunostimulation. We achieved this aim with the use of conventional anti-inflammatory agents and, more efficiently, with a selective p38 inhibitor supporting the suggestion that a p38-dependent inflammatory reaction underlies the phenomenon of tumor immunostimulation. In clinical trials for advanced cancer, immune-checkpoint inhibitors and other immunological approaches only demonstrated beneficial effects in a small cluster of patients (2, 30, 47). We suggest that these patients might for some reason have been unable to mount a significant inflammatory response preventing the emergence of a state of tumor immunostimulation. The observation presented in this work that the immunostimulatory arm of the IR curve is not observed in Winn assays carried out in test mice that cannot mount an inflammatory response, seems to support that suggestion. Similarly, the low incidence of spontaneous tumors exhibited by NSG mice might be attributed to their low ability to generate tumor immunostimulation associated with their defective capacity to mount both a specific antitumor IR and a macrophage-dependent inflammatory response (34). In fact, the therapeutic antitumor success (when it occurred) of BET (bromodomain and extra-terminal motif) inhibitors could be attributed, at least in part, to their ability to impair macrophage-mediated inflammation (50). A deeper understanding of the phenomenon of tumor immunostimulation together with the analysis of the genetic profile of tumor antigens aimed to distinguish between those that could induce strong, weak or tumor-stimulatory IRs could help to design improved immune-therapeutic approaches to cancer and eventually achieve the still elusive goal of eradicating well-established tumors by immunological means.

## Author Contributions

PC performed most of the experiment and helped in writing and correcting the manuscript. MV collaborated with the correction of the work and together with DM helped in the accomplishment of some experiments. PV and RM collaborated with immunohistochemistry experiments. AS collaborated with some experiments. OB helped in the design and realization of *in vivo* experiments. RR designed and supervised all experiments and drafted the manuscript. RP was the mentor, guiding and supporting the work.

## Conflict of Interest Statement

The authors declare that the research was conducted in the absence of any commercial or financial relationships that could be construed as a potential conflict of interest.

## References

[1] Prehn RT Main JM Immunity to methylcholanthrene-induced sarcomas. J Natl Cancer Inst (1957) 18:769.tyjl–78.tyjl. 13502695

[2] Janeway CA Travers P Walport M Shlomchik MJ Manipulation of the immune response. In: Janeway CA, editor. Immunobiology. New York: Garland (2001). p. 566–77.

[3] Chiarella P Vulcano M Bruzzo J Vermeulen M Vanzulli S Maglioco A Anti-inflammatory pretreatment enables an efficient dendritic cell-based immunotherapy against established tumors. Cancer Immunol Immunother (2008) 57:701–18. 10.1007/s00262-007-0410-4 17962945 PMC11030084

[4] Wiedermann U Davis AB Zielinski CC. Vaccination for the prevention and treatment of breast cancer with special focus on Her-2/neu peptide vaccines. Breast Cancer Res Treat (2013) 138:1–12. 10.1007/s10549-013-2410-8 23340862

[5] Pardoll DM. The blockade of immune checkpoints in cancer immunotherapy. Nat Rev Cancer (2012) 12:252–64. 10.1038/nrc3239 22437870 PMC4856023

[6] Snyder A Wolchok JD Chan TA Genetic basis for clinical response to CTLA-4 blockade. N Engl J Med (2015) 372:783 10.1056/NEJMc1415938 25693024

[7] Prehn RT. The immune reaction as a stimulator of tumor growth. Science (1972) 176:170–1. 10.1126/science.176.4031.170 5014438

[8] Ichim CV. Revisiting immunosurveillance and immunostimulation: implications for cancer immunotherapy. J Transl Med (2005) 3:8. 10.1186/1479-5876-3-8 15698481 PMC549049

[9] Chiarella P Reffo V Bruzzo J Bustuoabad OD Ruggiero RA. Therapeutic anti-tumor vaccines: from tumor inhibition to enhancement. Clin Med Oncol (2008) 2:237–45. 21892285 10.4137/cmo.s538 PMC3161645

[10] Prehn RT Prehn LM Cancer immunotherapy by immunosuppression. Theor Biol Med Mod (2010) 7:45–53. 10.1186/1742-4682-7-45 PMC3018371 21159199

[11] Hewitt HB Blake ER Walder AS A critique of the evidence for active host defence against cancer based on personal studies of 27 murine tumors of spontaneous origin. Br J Cancer (1976) 33:241–59. 10.1038/bjc.1976.37 773395 PMC2024987

[12] Rogers LM Olivier AK Meverholz DK Dupuy AJ. Adaptive immunity does not strongly suppress spontaneous tumors in a *Sleeping Beauty* model of cancer. J Immunol (2013) 190:4393–9. 10.4049/jimmunol.1203227 23475219 PMC3622230

[13] Schreiber RD Old LJ Smyth MJ Cancer immunoediting: integrating immunity’s roles in cancer suppression and promotion. Science (2011) 331:1565–70. 10.1126/science.1203486 21436444

[14] Zheng L Zhou B Meng X Zhu W Zuo A Wang X A model of spontaneous mouse mammary tumor for human estrogen receptor and progesterone receptor-negative breast cancer. Int J Oncol (2014) 45:2241–9. 10.3892/ijo.2014.2657 25230850 PMC4215580

[15] Meuwissen R Berns A Mouse models for human lung cancer. Genes Dev (2005) 19:643–64. 10.1101/gad.1284505 15769940

[16] Ruggiero RA Bustuoabad OD Bonfil RD Meiss RP Pasqualini CD “Concomitant immunity” in murine tumours of non-detectable immunogenicity. Br J Cancer (1985) 51:37–48. 10.1038/bjc.1985.6 2981538 PMC1976815

[17] Bustuoabad OD Ruggiero RA DiGianni P Lombardi MG Belli C Camerano GV Tumor transition zone: a putative new morphological and functional hallmark of tumor aggressiveness. Oncol Res (2005) 15:169–82. 10.3727/096504005776367933 16050138

[18] Meiss RP Bonfil RD Ruggiero RA Pasqualini CD. Histologic aspects of concomitant resistance induced by nonimmunogenic murine tumors. J Natl Cancer Inst (1986) 76:1163–75. 3458952

[19] Ruggiero RA Bustuoabad OD Cramer P Bonfil RD Pasqualini CD. Correlation between seric antitumor activity and concomitant resistance in mice bearing nonimmunogenic tumors. Cancer Res (1990) 50:7159–65. 2224851

[20] Franco M Bustuoabad OD di Gianni PD Goldman A Pasqualini CD Ruggiero RA. A serum-mediated mechanism for concomitant resistance shared by immunogenic and non-immunogenic murine tumours. Br J Cancer (1996) 74:178–86. 10.1038/bjc.1996.335 8688319 PMC2074564

[21] Ruggiero RA Bruzzo J Chiarella P di Gianni PD Isturiz MA Linskens S Tyrosine isomers mediate the classical phenomenon of concomitant tumor resistance. Cancer Res (2011) 71:7113–24. 10.1158/0008-5472.CAN-11-0581 22084446

[22] Manthey CL Wang SW Kinney SD Yao Z. SB202190, a selective inhibitor of p38 mitogen-activated protein kinase, is a powerful regulator of LPS-induced mRNAs in monocytes. J Leukoc Biol (1998) 64:409–17. 9738669 10.1002/jlb.64.3.409

[23] Charan S Huegin AW Cerny A Hengartner H Zinkernagel RM Effects of cyclosporine A on humoral immune response and resistance against vesicular stomatitis virus in mice. J Virol (1986) 57:1139–44. 3005615 10.1128/jvi.57.3.1139-1144.1986 PMC252848

[24] Zhu H Bengsch JE Svoronos N Rutkowski MR Bitler BJ Allegrezza MJ BET bromodomain inhibition promotes anti-tumor immunity by suppressing PD-L1 expression. Cell Rep (2016) 16:2829–37. 10.1016/j.celrep.2016.08.032 27626654 PMC5177024

[25] Spranger S Koblish HK Horton B Scherle PA Newton P Gajewski TF Mechanism of tumor rejection with doublets of CTLA-4, PD-1/PD-L1, or IDO blockade involves restored IL-2 production and proliferation of CD8+ T cells directly within the tumor microenvironment. J Immunother Cancer (2014) 2:3 10.1186/2051-1426-2-3 24829760 PMC4019906

[26] Lee CH Wu CL Shiau AL. Toll-like receptor 4 signaling promotes tumor growth. J Immunother (2010) 33:73–82. 10.1097/CJI.0b013e3181b7a0a4 19952954

[27] Koul HK Pal M Koul S. Role of p38 MAP kinase signal transduction in solid tumors. Genes Cancer (2013) 4:342–59. 10.1177/1947601913507951 24349632 PMC3863344

[28] Copier J Dalgleish A Whole-cell vaccines: a failure or a success waiting to happen. Curr Opin Mol Ther (2010) 12:14–20. 20140812

[29] Yu P Rowley DA Fu YX Schreiber H. The role of stroma in immune recognition and destruction of well-established solid tumors. Curr Opin Immunol (2006) 18:226–31. 10.1016/j.coi.2006.01.004 16459066

[30] Vieweg J Su Z Dahm P Kusmartsev S Reversal of tumor-mediated immunosuppression. Clin Cancer Res (2007) 13:727–32. 10.1158/1078-0432.CCR-06-1924 17255301

[31] Cerliani JP Dalotto-Moreno T Compagno D Dergan-Dylon LS Laderach DJ Gentilini L Study of galectins in tumor immunity: strategies and methods. Methods Mol Biol (2015) 1207:249–68. 10.1007/978-1-4939-1396-1_16 25253145

[32] Warning JC McCracken SA Morris JC. A balancing act: mechanisms by which the fetus avoids rejection by the maternal immune system. Reproduction (2011) 141:715–24. 10.1530/REP-10-0360 21389077

[33] Siegel CT Schreiber K Meredith SC Beck-Engeser GB Lancki DW Lazarski CA Enhanced growth of primary tumor in cancer-prone mice after immunization against the mutant region of an inherited oncoprotein. J Exp Med (2000) 191:1945–56. 10.1084/jem.191.11.1945 10839809 PMC2213520

[34] Shultz LD Schweitzer PA Christianson SW Gott B Schweitzer IB Tennent B Multiple defects in innate and adaptive immunologic function in NOD/LtSz-scid mice. J Immunol (1995) 154:180–91. 7995938

[35] Stutman O Immunodepression and malignancy. Adv Cancer Res (1975) 22:261–422. 10.1016/S0065-230X(08)60179-7 766581

[36] Qin Z Blankenstein T A cancer immunosurveillance controversy. Nat Immunol (2004) 5:3–4. 10.1038/ni0104-3 14699396

[37] Enzler T Gillessen S Manis JP Ferguson D Fleming J Alt FV Deficiencies of GM-CSF and interferon gamma link inflammation and cancer. J Exp Med (2003) 197:1213–9. 10.1084/jem.20021258 12732663 PMC2193978

[38] Stewart THM Henderson R Grayson H Opetz G Reduced incidence of rectal cancer, in a population of 73,076 men and women chronically immunosuppressed. Clin Cancer Res (1997) 3:51–5. 9815537

[39] Stewart THM Immunological enhancement of breast cancer. Parasitology (1997) 115:141–53. 10.1017/S0031182097001832 9571699

[40] Radaelli E Hermans E Omodho L Francis A Borght S Marine JC Spontaneous post-transplant disorders in NOD.Cg-Prkdcscid II2 rgtm1 Sug/Jic Tac (NOG) mice engrafted with patient-derived metastatic melanoma. PLoS One (2015) 10(5):e0124974 10.1371/journal.pone.0124974 25996609 PMC4440639

[41] Prehn RT Prehn LM. The flip side of immune surveillance: immune dependency. Immunol Rev (2008) 222:341–56. 10.1111/j.1600-065X.2008.00609.x 18364013

[42] Klein G Sjögren HO Klein E Hellström KE Demonstration of resistance against methylcholanthrene-induced sarcomas in the primary autochtonous host. Cancer Res (1960) 20:1561–72. 13756652

[43] Schreiber H Cancer immunology. 7th ed In: Paul WL, editor. Fundamental Immunology. Philadelphia, PA: Lippincott, Williams & Wilkins (2013). p. 1215–6.

[44] Basombrío MA Prehn RT Immune status of autochthonous and adoptively protected mice toward spontaneous and chemically induced tumors. Cancer Res (1972) 32:2545–50. 5082598

[45] Leidner RS Aboulafia DM Recrudescent Kaposi’s sarcoma after initiation of HAART: a manifestation of immune reconstitution syndrome. AIDS Patient Care STDS (2005) 19:635–44. 10.1089/apc.2005.19.635 16232048

[46] Hammond WG Benfeld JR Tesluk H Johnson JR Teplitz RL Tumor progression by lung cancers growing in hosts of different immunocompetence. Cancer J (1995) 8:130–8.

[47] Parmiani G Maccalli C The early antitumor immune response is necessary for tumor growth: re-visiting Prehn’s hypothesis in the human melanoma system. Oncoimmunology (2012) 1:930–4. 10.4161/onci.21455 23162761 PMC3489749

[48] He Y Zha J Wang Y Liu W Yang X Yu P Tissue damage-associated ‘danger signals’ influence T cell responses that promote the progression of pre-neoplasia to cancer. Cancer Res (2013) 73:629–39. 10.1158/0008-5472.CAN-12-2704 23108142

[49] Hoesel B Schmidt JA The complexity of NF-κB signaling in inflammation and cancer. Mol Cancer (2013) 12:86 10.1186/1476-4598-12-86 23915189 PMC3750319

[50] Belkina AC Nikolajczyk BS Denis GV. BET protein function is required for inflammation: Brd2 genetic disruption and BET inhibitor JQ1 impair mouse macrophage inflammatory responses. J Immunol (2013) 190:3670–8. 10.4049/jimmunol.1202838 23420887 PMC3608815

